# Clonal dissemination of successful emerging clone *mecA*-MRSA t304/ST6 among humans and hedgehogs in the Helsinki metropolitan area in Finland

**DOI:** 10.1016/j.onehlt.2023.100516

**Published:** 2023-02-21

**Authors:** Johansson Venla, Al-Mustapha Ahmad, Heljanko Viivi, Lindholm Laura, Salmenlinna Saara, Sainmaa Sanna, Heikinheimo Annamari

**Affiliations:** a Department of Food Hygiene and Environmental Health, Faculty of Veterinary Medicine, University of Helsinki, Agnes Sjöberginkatu 2, PO Box 66, 00790, Helsinki, Finland; b Expert Microbiology Unit, Department of Health Security, Finnish Institute for Health and Welfare, Mannerheimintie 166, PO Box 30, 00271, Helsinki, Finland; c Korkeasaari Zoo, Mustikkamaanpolku 12, PO Box 1000, 0081 Helsinki, Finland; d Microbiology Unit, Finnish Food Authority, Mustialankatu 3, PO Box 200, 00790 Helsinki, Finland

**Keywords:** Methicillin-resistant *Staphylococcus aureus*, Wild animals, Antimicrobial resistance, Human–wildlife interface, t304, Hedgehog

## Abstract

Methicillin-resistant *Staphylococcus aureus* (MRSA) carrying *mecC* gene (*mecC*-MRSA) is frequently reported among European hedgehogs (*Europeaus erineaus*) due to co-evolutionary adaptation to dermatophyte infection in European hedgehogs. The occurrence of MRSA in European hedgehogs in Finland is unknown. Consequently, we investigated the occurrence of MRSA in wild hedgehogs from urban Helsinki metropolitan area in 2020–2021 and applied whole genome sequencing (WGS) to further characterize the studied isolates and compared them with human clinical MRSA isolates. Altogether 115 dead hedgehogs were screened for MRSA using selective cultivation methods. Presumptive MRSA isolates were tested for antimicrobial susceptibility and confirmed MRSA isolates were further characterized by spa-typing and WGS. Hedgehog derived MRSA isolates were compared with clinical human MRSA isolates using core genome multilocus sequence typing (cgMLST). In total MRSA was recovered from 11 out of 115 (10%) hedgehogs. Among these four different spa types (t304; *n* = 4, t8835; n = 4, t5133; *n* = 2 and t622; *n* = 1) and three different sequence types (STs) (ST6; *n* = 6, ST7663; n = 4 and ST2840; n = 1) were identified. From the studied MRSA isolates seven harboured the *mecA* gene (*mecA*-MRSA) and four were identified as *mecC*-MRSA. All *mecA*-MRSA isolates carried immune evasion cluster genes, and one isolate was positive for Panton-Valentine leukocidin. cgMLST comparison revealed close genetic relatedness among three hedgehog and two human *mecA*-MRSA isolates all belonging to t304/ST6. Our results suggest a clonal dissemination of a successful MRSA clone among humans and hedgehogs. Further studies are warranted to investigate the sources and dissemination of such clone in urban environments. We observed a relatively low occurrence of mecC-MRSA in Finnish hedgehogs.

## Highlights

• The occurrence of MRSA in wild hedgehogs from the urban Helsinki metropolitan area was investigated.
• Multiple *mec*A-MRSA isolates harbouring human-specific markers were identified.
• cgMLST analysis revealed clonal dissemination of *mec*A-MRSA t304/ST6 among humans and hedgehogs.
• The occurrence of *mec*C MRSA was lower compared with previous studies done on wild hedgehogs.

## Graphical abstract

**Figure:**
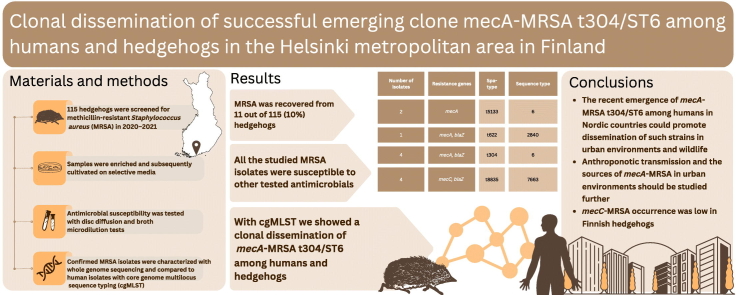

## 1 Introduction

Methicillin-resistant *Staphylococcus aureus* (MRSA) is a Gram-positive bacteria with zoonotic potential, responsible for approximately 150,000 infections every year in the European Union (EU), resulting in >7000 attributable deaths [1]. Initially emerging in the 1960s soon after methicillin was introduced for clinical use, MRSA was primarily associated with hospital infections [2]. Decades later, in the late 1990s, MRSA was demonstrated to be responsible for community-related infections [3] and in recent years, specific clonal lineages of MRSA have been associated with livestock [4]. Methicillin resistance in *S. aureus* is caused by the production of penicillin-binding protein 2, encoded by *mec* genes *mecA* (*mecA*-MRSA) and *mecC* (*mecC*-MRSA) [5,6]. Mobilized readily by staphylococcal cassette chromosome *mec* (SCC*mec*) [7], *mec* genes render MRSA strains resistant to most β-lactam antibiotics.

In 2011, a *mecA* homologue *mecA*_LGA251_ (also known as *mecC*) was described in MRSA strains carried by dairy cattle and later by humans [8,9]. While *mecC*-MRSA occurs only occasionally in humans [10] it is widely distributed among different domesticated animal and wildlife species [4,11] and is especially prevalent in European hedgehogs (*Erinaceus europaeus*) [[12], [13], [14], [15]]. Studies spanning from the 1960s have demonstrated the co-occurrence of penicillin-like substance-producing dermatophyte species *Trichophyton erinacei* with penicillin-resistant MRSA, and later specifically *mecC*-MRSA, in hedgehogs [15,16]. These findings led to a hypothesis of hedgehogs being a primary host for *mecC*-MRSA. Subsequently, Larsen et al. [13] demonstrated that *mecC*-MRSA predates the modern use of antimicrobials in European hedgehogs as a result of co-evolutionary adaptation to dermatophyte infection.

Similarly, evidence from numerous studies suggests that carriage of antimicrobial-resistant bacteria (ARB) and antimicrobial-resistant genes (ARGs) in wildlife is not necessarily due to selection pressure from the clinical use of antibiotics but is instead a sign of anthropogenic pollution [17]. The spillover of ARB/ARG into wildlife populations may occur through various anthropogenic sources, such as waste disposal, wastewater management or agricultural activity [[18], [19], [20]] and exposure is more likely for species living near humans, as demonstrated by a recent study in which a high prevalence of multidrug-resistant Enterobacteriaceae in European hedgehogs was noted in highly populated areas of Spain [21].

The occurrence of MRSA in wild hedgehogs has been well documented in some Nordic countries; however, no reports from Finland exist. In this context, we studied MRSA in wild hedgehogs from the urban Helsinki metropolitan area in 2020–2021, characterized the isolates by whole genome sequencing (WGS) and compared them with human clinical MRSA isolates to further understand the epidemiology of the studied isolates.

## 2 Materials and methods

The study material consisted of 115 (54 males, 35 females and 26 not identified) deceased or euthanized wild hedgehogs from the Helsinki metropolitan area, Finland, brought to the Korkeasaari Zoo's Wildlife Hospital during 2020 and 2021 due to injuries or other disease symptoms. Some of the hedgehogs (*n* = 51) were already deceased or euthanized outside the hospital premises, and some were kept in the wildlife hospital before dying naturally or being euthanized (*n* = 38). For some animals this information was missing (*n* = 26). Each hedgehog was placed in a plastic bag and kept in a − 20 °C freezer until transportation to the laboratory. Ethical approval was not required according to the Nature Conservation Act (1096/1996 §40,49). The rest of the metadata are presented in Supplementary Table 1. Data from human clinical MRSA isolates originate from the national infectious disease register. Clinical microbiology laboratories in Finland are required to send all MRSA findings to the Finnish Institute for Health and Welfare (THL) for *spa* typing. WGS is performed for a subset of these, for example, invasive isolates, isolates linked to outbreaks or those selected for specific projects. s*pa* typing is performed to all MRSA isolates sent to THL, yearly on approximately 1500 MRSA isolates, and approximately 1600 isolates have been typed by WGS since 2015.

### 2.1 Isolation, species confirmation and antimicrobial susceptibility testing of methicillin-resistant *Staphylococcus aureus*

To determine the carriage of MRSA, swab samples were obtained from each hedgehog from the nostril, buccal cavity, and perineum just after thawing. Each swab was enriched in 9 ml Müller Hinton broth (Sigma-Aldrich, Saint Louis, MO, USA) with 6.5% NaCl and incubated at 37 °C for 16–24 h. Subsequently, a 10 μl loopful of the suspension was spread onto CHROMagar™ MRSA (CHROMagar, Paris, France) and the plate was incubated at 37 °C for 16–24 h. Up to two presumptive *S. aureus* colonies from each plate with visible growth were subcultured on blood agar plates (Oxoid, Basingstoke, UK) and incubated at 37 °C for 22–24 h. Species identification was done with matrix-assisted laser desorption/ionization time-of-flight mass spectrometry (MALDI-TOF) (Bruker MALDI Biotyper Microflex LT, Bruker Daltonik GmbH, Bremen, Germany) by picking and inoculating a freshly grown overnight colony onto MALDI-TOF target plate. Spots were allowed to dry and subsequently overlaid with 1 μl of matrix (α-cyano-4-hydroxycinnamic acid) (Bruker Daltonik, GmbH, Bremen, Germany). A score value of 2.0–3.0 was considered high confidence and was set as the criteria.

For presumptive MRSA strains, antimicrobial susceptibility was tested with the disc diffusion method using 30 μg cefoxitin discs (Rosco Diagnostica, Taastrup, Denmark). Minimum inhibitory concentration values were determined using a Sensititre™ EUSTAPF plate (ThermoFisher Scientific, East Grinstead, UK) (Table 1). The results were interpreted according to European Committee on Antimicrobial Susceptibility Testing epidemiological cut-off values [22]. *S. aureus* ATCC 25178 was used as a negative reference strain. Isolates resistant to cefoxitin were stored at −80 °C for further analysis.

**Table 1:** Zone of inhibition and minimum inhibitory concentration (MIC) of antimicrobials for 11 methicillin-resistant *Staphylococcus aureus* isolates from hedgehogs.

Isolate	Zone of inhibition (mm)^a^	MIC (mg/l)^b^																		
Antibiotic^c^	CFO30	FOX	CPT	CLI	DAP	ERY	FUS	GEN	LEVO	LZD	MXF	MUP	NOR	RIF	TEI	TLA	TET	TOB	SXT	VAN
ECOFF^d^	22	≤4	≤(1)	≤0.25	≤1	≤1	≤0.5	≤2	≤0.5	≤4	≤0.25	≤1	≤(4)	≤0.03	≤2	≤1	≤1	≤2	≤(0.25)	≤2
D884	**9.7**	**8**	<0.5	<0.12	<0.5	<0.25	<0.5	<0.25	0.5	<2	<0.25	<0.5	<4	<0.03	<1	<0.03	<0.5	<0.25	<1	0.5
D888	**11.7**	**8**	<0.5	<0.12	<0.5	<0.25	<0.5	<0.25	0.5	<2	<0.25	<0.5	<4	<0.03	<1	<0.03	<0.5	<0.25	<1	0.5
D1	**11**	**8**	<0.5	<0.12	<0.5	0.25	<0.5	<0.25	0.5	2	<0.25	<0.5	<4	<0.03	<1	0.03	<0.5	0.25	<1	0.5
D2	**11.8**	**8**	<0.5	<0.12	<0.5	0.25	<0.5	<0.25	0.5	<2	<0.25	<0.5	<4	<0.03	<1	0.03	<0.5	<0.25	<1	0.5
D3	**0**	**8**	<0.5	<0.12	<0.5	0.25	<0.5	<0.25	0.5	2	<0.25	<0.5	<4	<0.03	<1	0.03	<0.5	0.25	<1	0.5
D4	**0**	**8**	<0.5	<0.12	<0.5	0.25	<0.5	0.25	0.5	2	<0.25	<0.5	<4	<0.03	<1	0.03	<0.5	<0.25	<1	0.5
D5	**0**	**8**	<0.5	<0.12	<0.5	0.25	<0.5	0.25	0.5	2	<0.25	<0.5	<4	<0.03	<1	0.03	<0.5	<0.25	<1	0.5
D6	**11**	**8**	<0.5	<0.12	<0.5	0.25	<0.5	<0.25	0.5	2	<0.25	<0.5	<4	0.03	<1	0.03	<0.5	<0.25	<1	1
D7	**0**	**8**	<0.5	<0.12	<0.5	0.25	<0.5	<0.25	0.5	2	<0.25	<0.5	<4	<0.03	<1	0.03	<0.5	<0.25	<1	0.5
D8	**10**	**8**	<0.5	<0.12	<0.5	0.25	<0.5	<0.25	0.5	2	<0.25	<0.5	<4	<0.03	<1	<0.03	<0.5	<0.25	<1	0.5
D411	**0**	**8**	0.5	<0.12	<0.5	<0.25	<0.5	<0.25	0.5	<2	<0.25	<0.5	<4	<0.03	<1	<0.03	<0.5	<0.25	<1	0.5

a Zones of inhibition in bold lettering are under the epidemiological cut-off value (ECOFF).

b MICs in bold lettering are above the ECOFF cut-off value.

c CFO30, cefoxitin (30 μg); FOX, cefoxitin; CPT, ceftaroline; CLI, clindamycin; DAP, daptomycin; ERY, erythromycin; FUS, fusidate; GEN, gentamicin; LEVO, levofloxacin; LZD, linezolid; MXF, moxifloxacin; MUP, mupirocin; NOR, norfloxacin; RIF, rifampin; TEI, teicoplanin; TLA, telavancin; TET, tetracycline; TOB, tobramycin; SXT, trimethoprim/sulfamethoxazole; VAN, vancomycin.

d Tentative ECOFFs are indicated inside brackets.

### 2.2 Confirmation of methicillin-resistant *Staphylococcus aureus* and *spa* typing

Confirmation of MRSA was done by in-house multiplex polymerase chain reaction (PCR) targeting *mec* using the method developed by Stegger et al. [23] following the protocol recommended by the EU Reference Laboratory [24]. In addition, bacterial isolates were characterized with a simplex polymerase chain reaction (PCR) targeting the *spa* gene, following the method developed by Shopsin et al. [25]. The PCR products (300–400 bp) were sent for Sanger sequencing (Institute of Biotechnology, Helsinki, Finland) and the *spa* types were assigned using the *spa*-typing task template in Ridom SeqSphere+8.3 [26].

### 2.3 Whole genome sequencing and bioinformatic analyses

Bacterial DNA extraction and purification was done for MRSA isolates with a Qiagen DNeasy Blood & Tissue Kit (Qiagen, Valencia, CA, USA) according to the manufacturer's instructions. DNA quality was assessed with a NanoDrop ND-1000 spectrophotometer (Thermo Fischer Scientific, Wilmington, Delaware, USA) and quantity with a Qubit 2.0 Fluorometer (Invitrogen Life Technologies, Carlsbad, CA, USA). The extracted DNA was stored at −80 °C prior to sequencing. The library preparation was done with a NEBNext Ultra DNA Library Prep Kit (Illumina, San Diego, CA, USA) and the sequencing was performed with the Illumina NovaSeq 6000 platform (Illumina, San Diego, CA, USA) with 2 × 150 bp paired-end reads.

The quality of the raw reads were assessed with MultiQC 1.12 [27] and reads were trimmed using Trimmomatic 0.39 [28]. Draft genomes were obtained by using SPAdes 3.15.0 [29]. ARGs, plasmid replicons, virulence genes, SCC*mec* elements and multilocus sequence types (STs) were determined from assembled reads through the Center for Genomic Epidemiology tools (Technical University of Denmark, Denmark) with ResFinder 4.1 [30], PlasmidFinder 2.1 [31], VirulenceFinder [32], SCC*mec*Finder 1.2 [33] and MLST 2.0 [34] according to the server's default parameters. The immune evasion cluster (IEC) types were assigned according to the presence of genes associated with the IEC system [35]. New multilocus sequence typing (MLST) allele combinations were submitted to the PubMLST database [36]. The MLST clonal complexes (MLST-CCs) for MRSA isolates were determined using the PubMLST database [36].

### 2.4 Genomic comparison of MRSA strains

Subsequently, raw reads of the hedgehog MRSA isolates were sent to THL for further investigation. Ridom SeqSphere+8.5.1 [26] was used to perform a core genome MLST (cgMLST) analysis at THL to compare the studied MRSA isolates from hedgehogs (*n* = 11) with all the sequenced human MRSA isolates from the Finnish National Infectious Diseases Register (n = 1,600). Raw reads from both human and hedgehog isolates were de novo assembled using the Velvet algorithm v1.1.04 [37] and the public cgMLST scheme for *S. aureus* was used with default parameters [38].

## 3 Results

### 3.1 Isolation and detection of MRSA

According to species confirmation 11 out of 32 (34%) presumptive *S. aureus* colonies were confirmed as *S. aureus.* Consequently, *S. aureus* was recovered from 11 out of 115 (10%) hedgehogs and all isolates were resistant to cefoxitin and harboured either *mecA* or *mecC* gene, i.e., the isolates were considered to be MRSA (Table 1, Table 2 and Supplementary Table 1). All the studied MRSA isolates were susceptible to other tested antimicrobials. (Table 1).

**Table 2:** Background information and genomic characteristics for the 11 methicillin-resistant *Staphylococcus aureus* isolates from hedgehogs.

Isolate	City	Residential district	Year	Resistance genes	SCC*mec* type^a^	Multilocus sequence type	Clonal complex (CC)	*spa* type	Virulence genes	Immune evasion cluster type	Plasmid replicons
D1	Vantaa	Louhela	2020	*mecA*	IVa(2B)	6	CC5	t5133	*aur*, *splA*, *splB*, *splE*, *hlgA*, *hlgB*, *hlgC*, *lukD*, *lukE*, *sea*, *sak*, *scn*	E	–
D2	Kirkkonummi	Kuusela	2020	*mecA*	IVa(2B)	6	CC5	t5133	*sak*, *scn*, *hlgA*, *hlgB*, *hlgC*, *lukD*, *lukE*, *sea*, *aur*, *splA*, *splB*, *splE*	E	rep20, rep7c
D3	Helsinki	Vuosaari	2020	*mecA*, *bla*Z	IVc(2B)	2840	CC8	t622	*sak*, *scn*, *aur*, *splA*, *splB*, *splE*, *hlgA*, *hlgB*, *hlgC*, *lukD*, *lukE*, *lukF-PV*, *lukS-PV*	E	–
D4	Helsinki	Malmi	2020	*mecA*, *bla*Z	IVc(2B)	6	CC5	t304	*hlgA*, *hlgB*, *hlgC*, *lukD*, *lukE*, *sea*, *aur*, *splA*, *splB*, *splE*, *sak*, *scn*	D	rep5a, rep16
D5	Helsinki	Munkkivuori	2020	*mecA*, *bla*Z	IVa(2B)	6	CC5	t304	*sak*, *scn*, *aur*, *splA*, *splA*, *splB*, *splE*, *hlgA*, *hlgB*, *hlgC*, *lukD*, *lukE*, *sea*	D	rep5a, rep16
D6	Helsinki	Malmi	2020	*mecA*, *bla*Z	IVa(2B)	6	CC5	t304	*aur*, *splA*, *splB*, *splE*, *sak*, *scn*, *hlgA*, *hlgB*, *hlgC*, *lukD*, *lukE*, *sea*	D	rep5a, rep16
D7	–	–	2020	*mecA*, *bla*Z	IVa(2B)	6	CC5	t304	*hlgA*, *hlgB*, *hlgC*, *lukD*, *lukE*, *sea*, *sak*, *scn*, *aur*, *splA*, *splB*, *splE*	D	rep5a, rep16
D8	Helsinki	Etu-Töölö	2020	*mecC*, *bla*Z	XI(8E)	7663	–	t8835	*aur*, *splA*, *splB*, *splE*, *hlgA*, *hlgB*, *hlgC*, *lukD*, *lukE*, *sec*, *seg*, *sei*, *sel*, *sem*, *sen*, *seo*, *seu*, *tst*	–	–
D884	Helsinki	Landbo	2020	*mecC*, *bla*Z	XI(8E)	7663	–	t8835	*hlgA*, *hlgB*, *hlgC*, *lukD*, *lukE*, *sec*, *seg*, *sei*, *sel*, *sem*, *sen*, *seo*, *seu*, *tst*, *aur*, *splA*, *splB*, *splE*	–	–
D888	Helsinki	Etu-Töölö	2020	*mecC*, *bla*Z	XI(8E)	7663	–	t8835	*aur*, *splA*, *splB*, *splE*, *hlgA*, *hlgB*, *hlgC*, *lukD*, *lukE*, *sec*, *seg*, *sei*, *sel*, *sem*, *sen*, *seo*, *seu*, *tst*	–	–
D411	Helsinki	Meilahti	2020	*mecC*, *bla*Z	XI(8E)	7663	–	t8835	*aur*, *splA*, *splA*, *splB*, *splE*	–	–

a SCC*mec*, staphylococcal cassette chromosome *mec*.

### 3.2 s*pa* typing

Among the 11 studied isolates, four different *spa* types, namely t304 (*n* = 4), t8835 (n = 4), t5133 (*n* = 2) and t622 (*n* = 1) were identified (Table 2).

### 3.3 Whole genome sequencing and bioinformatic analyses

Among the 11 isolates, 4 were carrying the *mecC* and 7 the *mecA* gene. Furthermore, *blaZ* was carried by 8 out of 11 (73%) isolates (Table 2). The 11 studied isolates belonged to three different multilocus STs, namely ST6 (*n* = 6), ST7663 (n = 4) and ST2840 (n = 1). STs could be further classified into two different clonal groups (MLST-CC): ST6 belonged to CC5 and ST2840 belonged to CC8. There was no assigned MLST-CC for ST7663.

All the t304/ST6 isolates (n = 4) carried *rep*5a and *rep*16, and the t5133/ST6 isolate carried the *rep*20 and *rep*7c replicons. All the *mecA*-MRSA isolates were IEC-positive, three of them being type E (carrying the *scn* and *sak* genes) and four of them being type D (carrying the *scn*, *sak* and *sea* genes). Moreover, t622/ST2840 was determined to be PVL-positive, harbouring the *lukF-PV* and *lukS-PV* genes. Other virulence genes are presented in Table 2. All the *mecA*-MRSA isolates had SCC*mec* type IV, including subvariants Va(2B) and Vc(2B). All the *mecC*-MRSA isolates had SCC*mec* type XI(8E).

### 3.4 Genomic comparison of MRSA strains

**Fig. 1:**
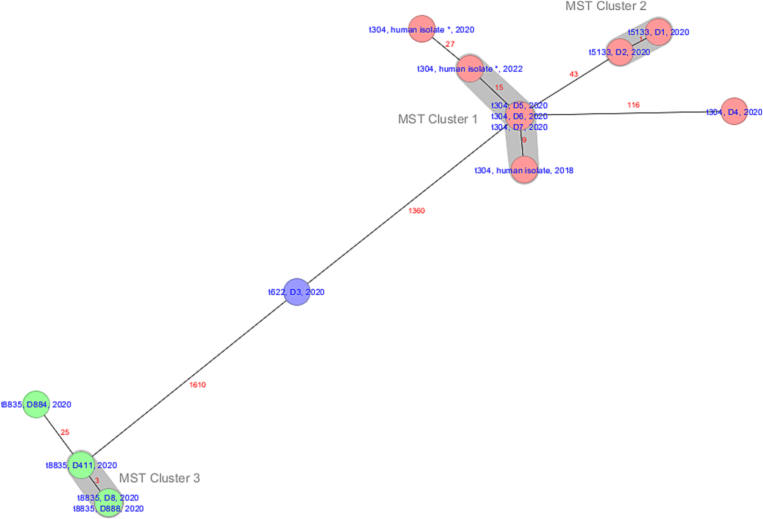
Minimum spanning tree of all hedgehog MRSA (*n* = 11) and three human isolates illustrating their genetic relationship based on up to 1712 cgMLST target genes, pairwise ignoring missing values. The numbers represent the allelic differences between isolates. The isolates are denoted with *spa* type, isolate and isolation year. Each colour represents a sequence type (ST). Red: ST6, blue: ST2840 and green: ST7663. Asterisk sign denotes that the isolates originate from the same person. (For interpretation of the references to colour in this figure legend, the reader is referred to the web version of this article.)

**Supplementary Fig. 1:**
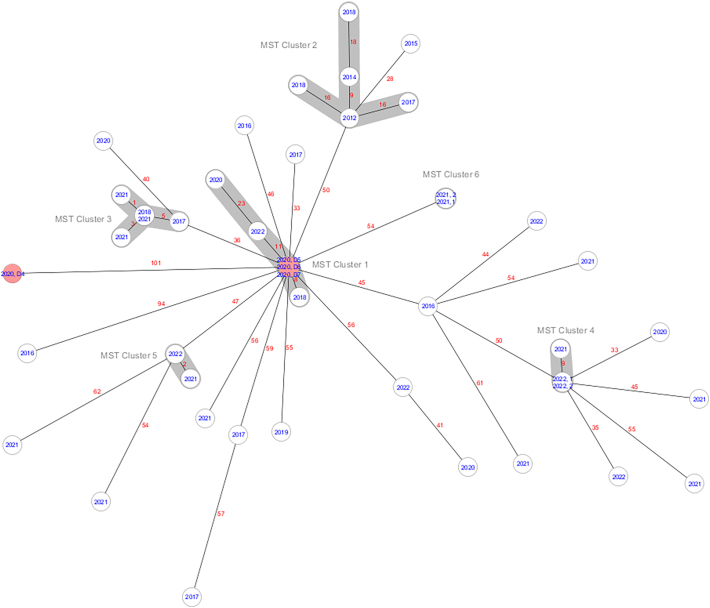
Minimum spanning tree of all hedgehog (n=4) and human (n=41) t304/ST6 mecA-MRSA isolates illustrating their genetic relationship based on up to 1,512 cgMLST target genes, with no missing values. The numbers represent the allelic differences between isolates. The isolates are denoted with isolation year. Red and white colour represents hedgehog and human derived isolates, respectively.

We identified three clusters based on a defined cluster threshold of 24 allelic differences (Fig. 1) [38]. Two human MRSA t304/ST6 isolates, one from 2018 (Western part of Finland) and another one from 2022 (Helsinki metropolitan area) showed close relatedness, with 9 and 15 allelic differences respectively compared with identical hedgehog strains D5, D6 and D7 (Cluster 1). The third human MRSA t304/ST6 isolate from 2020 had 27 allelic differences compared with the first cluster. The second cluster (Cluster 2) included two t5133/ST6 *mecA*-MRSA hedgehog isolates, D1 and D2, with one allele difference, and the last cluster (Cluster 3) included four t8835/ST7663 *mecC*-MRSA hedgehog isolates, from which D8 and D888 were identical, while D411 had three and D884 had 25 allelic differences compared with the identical isolates. A distance matrix of all the t304/ST6 isolates (*n* = 45) from 2012 to 2022 showed that the range of allelic differences between two isolates was 0–135 with a median of 66 allelic differences (Supplementary Fig. 1 and supplementary Table 3).

## 4 Discussion

In this study we show a clonal dissemination of a successful clone, t304/ST6 *mecA*-MRSA, among humans and hedgehogs, as evidenced by cgMLST analysis. In the absence of direct epidemiological linkage of the studied hedgehog and human isolates, we argue that the plausible direction for the transmission of IEC-positive *mecA*-MRSA clones is from humans to hedgehog (spillback event) through contaminated environment. As demonstrated in previous studies, spillback of antimicrobial resistance from humans to wildlife may be enabled by spatial proximity [17,18,20,21] and hedgehogs commonly inhabit areas with high human activity, providing them with food sources and nesting areas but also opportunities for indirect and physical contact with humans and their waste [39]. However, our data cannot rule out the directionality from hedgehogs to humans. Two of the hedgehogs harbouring identical t304/ST6 *mec*A-MRSA strains (D5 and D6) originated from different residential areas in Helsinki and were treated at different times in the wildlife hospital. This could suggest for the possibility of nosocomial spread of the clone through contaminated surfaces and hospital staff in the wildlife hospital. In contrast, available evidence from previous studies suggests that most of the hedgehogs acquire MRSA outside rescue facilities and that occurrence of MRSA is only slightly lower in hedgehogs that died in the wild compared with hedgehogs that stayed in rescue facilities [12,13]. Moreover, our data point out the possibility of two separate introductions of t304/ST6 *mecA*-MRSA to the local hedgehog population, as demonstrated by the allelic difference of 116 and different SCCmec types between isolates D4 and D6, originating from hedgehogs collected in the same month and area. Nevertheless, it is difficult to assess the directionalities of these transmissions without more extensive knowledge on the occurrence of this clone in animals and the environment.

The t304/ST6 *mecA*-MRSA clone has recently emerged and t304 has become one of the most common *spa* types in Northern Europe among humans, concurrent with the influx of refugees from the Syrian civil war to Europe [40,41]. In addition to core genome allelic similarity, the studied t304/ST6 *mecA*-MRSA isolates had similar genomic features common to the broader t304/ST6 population in Nordic countries, including *rep*5a and *rep*16 plasmid replicons and SCC*mec* type IVa [40]. Moreover, all the studied t304/ST6 *mecA*-MRSA isolates were PVL-negative. According to the literature, t304/ST6 *mecA*-MRSA has been described from broiler meat in Germany [42], pigs in Sri Lanka [43], cheese in Egypt [44] and from a feral cat in Poland [45]. Moreover, it was described as the predominant clone isolated from two separate foodborne outbreaks in China [46]. Indeed, food products contaminated with t304/ST6 *mecA*-MRSA harbouring staphylococcal enterotoxins, such as *sea*, could be source for foodborne illnesses. The remarkable ability of *S. aureus* to adapt to or colonize multiple hosts calls for future studies to further investigate possible t304/ST6 *mecA*-MRSA sources and host interactions leading to dissemination, and possible development of secondary reservoirs in urban environments.

Another finding of our study also indicates a possible spillback of MRSA from humans to hedgehogs. In addition to t304/ST6, our study found three other *mecA*-MRSA isolates, belonging to t5133/ST6/CC5 (*n* = 2) and PVL-positive t622/ST2840/CC8 (*n* = 1). All these isolates had markers of human adaptations, namely the presence of human-specific IEC genes, *scn* and *sak* [35]. Moreover, these isolates harboured the SCC*mec* type IV element (subtypes a and c) commonly associated with community-associated MRSA [3]. Altogether, these findings suggest that the studied isolates originate from human-adapted strains. In contrast to *mecC*-MRSA, *mecA*-MRSA is detected in wildlife only occasionally [11] and there are only two reported cases of *mecA*-MRSA in hedgehogs (one from Spain and another from Hungary), belonging to CC1 and CC45, respectively [47,48]. There seems to be a compelling reason to argue that *mecA*-MRSA isolates in wildlife originate from humans. On a broader scale, however, the significance of wildlife species as *mecA* reservoirs remains unclear due to the fragmented nature of the reported cases and lack of comprehensive investigations into the possible anthropogenic and environmental factors facilitating the dissemination of such strains. We acknowledge these shortcomings in our study too and suggest further epidemiological studies to unravel why certain *mecA*-MRSA strains occur among urban wildlife.

We identified only four (3.5%, 4/115) *mecC*-MRSA isolates among the studied hedgehogs. Although this result differs considerably from those in other Nordic countries [12,14,15], it is consistent with a recent study that reported a low occurrence of *mecC*-MRSA in hedgehogs in Hungary [48]. Furthermore, *mecC*-MRSA has not been detected in hedgehogs in Greece, Romania, Italy, France and Spain [13]. This could indicate that, initially, particular *mecC*-MRSA lineages were not distributed evenly among European hedgehog populations during the postglacial expansion in Europe, despite long-distance dispersal events across some countries, as delineated by Larsen et al. [13]. However, the new MLST allele combination ST7663, being a single-locus variant of ST1943, is at least by definition part of CC1943, which is one of the most successful and oldest *mecC*-MRSA clones in Europe and initially emerged in hedgehogs [13]. This shows that potentially some descendants of the CC1943 lineage occur among hedgehogs in the Helsinki metropolitan area, although at low frequencies. In Finland, only sporadic cases of human *mecC*-MRSA occur and, presumably, the prevalence of *mecC*-MRSA is also low in Finnish livestock, as only one *mecC*-MRSA isolate, belonging to t3256/ST130, has been described from a mastitis sample from a cow [49]. However, *mecC*-MRSA could also be under-reported, since most of the clinical samples from livestock are not routinely isolated and clinical laboratories might have methods that are not sensitive enough to detect *mecC*-MRSA [50]. Indeed, the most plausible reason for the low occurrence of *mecC*-MRSA in this study could be due to methodological reasons as most of the chromogenic agars used in clinical settings are optimized to detect *mecA*-MRSA and there might be notifiable differences in how commercial chromogenic agars detect *mecC*-MRSA isolates [50]. In this study, we used a selective chromogenic agar, which could have a low sensitivity for *mecC-*MRSA, and therefore a chromogenic agar with known performance on *mec*C-MRSA should be used in the future studies. Moreover, we acknowledge that Finnish hedgehogs should be sampled at a wider geographical scale, representing environments from rural to semi-rural areas, and during different seasons. Most of the hedgehogs in this study were collected during summer months, and previous studies have shown that *T. erinacei* is especially prevalent in hedgehogs during winter [51]. This could simultaneously affect the occurrence of *mecC*-MRSA carriage in hedgehogs. However, a recent study by Dube et al. [15] managed to recover a high prevalence of *mecC*-MRSA from Swedish hedgehogs that were sampled from spring to early autumn, although the prevalence of *T. erinacei* in these samples remained low. Moreover, sampling only dead and frozen animals could affect the overall success of the isolation of MRSA strains, although previous studies have successfully recovered MRSA strains with similar sampling [12,14].

## 5 Conclusions

In conclusion, we report a possible spillback event of the *mecA*-MRSA t304/ST6 strain from humans to hedgehogs in Finland. This finding underlies the relevance of integrating antimicrobial resistance surveillance in wildlife to capture the complex epidemiology of MRSA. Although anthroponotic transmission of *mecA*-MRSA to wildlife seems to occur only sporadically, urbanized environments might escalate this phenomenon, especially among wild animals that live in close proximity to humans. To prevent the possible development of secondary reservoirs and preserve the health of humans, further studies assessing transmission and the sources of *mecA*-MRSA clones in urban environments are needed. The reason for the low occurrence of *mecC*-MRSA in Finnish hedgehogs remains unanswered.

The following are the supplementary data related to this article.

Supplementary Table 1 Background information for all the studied hedgehogs (n = 115). Supplementary Table 1

Supplementary Table 2 Accession numbers for the raw reads disposed of at the European Nucleotide Archive (ENA) at EMLBL-EBI. Supplementary Table 2

Supplementary Table 3 The cgMLST distance matrix of all the hedgehog (n=4) and human (n=41) t304/ST6 isolates. Isolate identification numbers starting with D are derived from hedgehogs, and those starting with H are derived from humans. Supplementary Table 3

## Funding

This work was supported by the 10.13039/501100002341Academy of Finland (grant number 339417).

## Ethical approval

Not required.

## Author statement

The authors have read and approved the revised version submitted.

## CRediT authorship contribution statement

**Johansson Venla:** Conceptualization, Methodology, Investigation, Software, Data curation, Formal analysis, Writing – original draft, Writing – review & editing, Visualization. **Al-Mustapha Ahmad:** Conceptualization, Methodology, Investigation, Writing – review & editing. **Heljanko Viivi:** Investigation, Writing – review & editing. **Lindholm Laura:** Conceptualization, Formal analysis, Resources, Writing – review & editing, Visualization. **Salmenlinna Saara:** Conceptualization, Resources, Writing – review & editing. **Sainmaa Sanna:** Conceptualization, Resources, Writing – review & editing. **Heikinheimo Annamari:** Conceptualization, Supervision, Project administration, Writing – review & editing.

## Declaration of Competing Interest

None.

## Data Availability

Raw reads of the hedgehog isolates have been disposed of at the European Nucleotide Archive (ENA) at EMBL-EBI under study accession number PRJEB57870. Sample accession numbers are provided in Supplementary Table 2.

## References

[1] Cassini A., Högberg L.D., Plachouras D., Quattrocchi A., Hoxha A., Simonsen G.S., Colomb-Cotinat M., Kretzschmar M.E., Devleesschauwer B., Cecchini M., Ouakrim D.A., Oliveira T.C., Struelens M.J., Suetens C., Monnet D.L., Strauss R., Mertens K., Struyf T., Catry B., Latour K., Ivanov I.N., Dobreva E.G., Tambic Andraševic A., Soprek S., Budimir A., Paphitou N., Žemlicková H., Schytte Olsen S., Wolff Sönksen U., Märtin P., Ivanova M., Lyytikäinen O., Jalava J., Coignard B., Eckmanns T., Abu Sin M., Haller S., Daikos G.L., Gikas A., Tsiodras S., Kontopidou F., Tóth Á., Hajdu Á., Guólaugsson Ó., Kristinsson K.G., Murchan S., Burns K., Pezzotti P., Gagliotti C., Dumpis U., Liuimiene A., Perrin M., Borg M.A., de Greeff S.C., Monen J.C., Koek M.B., Elstrøm P., Zabicka D., Deptula A., Hryniewicz W., Caniça M., Nogueira P.J., Fernandes P.A., Manageiro V., Popescu G.A., Serban R.I., Schréterová E., Litvová S., Štefkovicová M., Kolman J., Klavs I., Korošec A., Aracil B., Asensio A., Pérez-Vázquez M., Billström H., Larsson S., Reilly J.S., Johnson A., Hopkins S. (2019). Attributable deaths and disability-adjusted life-years caused by infections with antibiotic-resistant bacteria in the EU and the European Economic Area in 2015: A population-level modelling analysis. Lancet Infect. Dis..

[2] Jevons M.P. (1961). “Celbenin”—resistant staphylococci. Br. Med. J..

[3] Vandenesch F., Naimi T., Enright M.C., Lina G., Nimmo G.R., Heffernan H., Liassine N., Bes M., Greenland T., Reverdy M.-E., Etienne J. (2003). Community-acquired methicillin-resistant *Staphylococcus aureus* carrying Panton-valentine Leukocidin genes: worldwide emergence. Emerg. Infect. Dis..

[4] Zarazaga M., Gómez P., Ceballos S., Torres C. (2018). Staphylococcus Aureus.

[5] Fishovitz J., Hermoso J.A., Chang M., Mobashery S. (2014). Penicillin-binding protein 2a of methicillin-resistant *Staphylococcus aureus*. IUBMB Life.

[6] García-Álvarez L., Holden M.T., Lindsay H., Webb C.R., Brown D.F., Curran M.D., Walpole E., Brooks K., Pickard D.J., Teale C., Parkhill J., Bentley S.D., Edwards G.F., Girvan E.K., Kearns A.M., Pichon B., Hill R.L., Larsen A.R., Skov R.L., Peacock S.J., Maskell D.J., Holmes M.A. (2011). Meticillin-resistant Staphylococcus aureus with a novel mecA homologue in human and bovine populations in the UK and Denmark: a descriptive study. Lancet Infect. Dis..

[7] Katayama Y., Ito T., Hiramatsu K. (2000). A new class of genetic element, staphylococcus cassette chromosome *mec*, encodes methicillin resistance in *Staphylococcus aureus*. Antimicrob. Agents Chemother..

[8] García-Álvarez L., Holden M.T., Lindsay H., Webb C.R., Brown D.F., Curran M.D., Walpole E., Brooks K., Pickard D.J., Teale C., Parkhill J., Bentley S.D., Edwards G.F., Girvan E.K., Kearns A.M., Pichon B., Hill R.L., Larsen A.R., Skov R.L., Peacock S.J., Maskell D.J., Holmes M.A. (2011). Meticillin-resistant *Staphylococcus aureus* with a novel mecA homologue in human and bovine populations in the UK and Denmark: a descriptive study. Lancet Infect. Dis..

[9] Shore A.C., Deasy E.C., Slickers P., Brennan G., O’Connell B., Monecke S., Ehricht R., Coleman D.C. (2011). Detection of Staphylococcal Cassette Chromosome *mec* Type XI Carrying Highly Divergent *mecA*, *mecI*, *mecR1*, *blaZ*, and *ccr* Genes in Human Clinical Isolates of Clonal Complex 130 Methicillin-Resistant *Staphylococcus aureus*. Antimicrob. Agents Chemother..

[10] Lozano C., Fernández-Fernández R., Ruiz-Ripa L., Gómez P., Zarazaga M., Torres C. (2020). Human mecC-carrying MRSA: clinical implications and risk factors. Microorganisms..

[11] Silva V., Capelo J.L., Igrejas G., Poeta P. (2020). Molecular epidemiology of Staphylococcus aureus lineages in wild animals in Europe: A review. Antibiotics..

[12] Rasmussen S.L., Larsen J., van Wijk R.E., Jones O.R., Berg T.B., Angen Ø., Larsen A.R. (2019). European hedgehogs (*Erinaceus europaeus*) as a natural reservoir of methicillin-resistant *Staphylococcus aureus* carrying mecC in Denmark. PLoS One.

[13] Larsen J., Raisen C.L., Ba X., Sadgrove N.J., Padilla-González G.F., Simmonds M.S.J., Loncaric I., Kerschner H., Apfalter P., Hartl R., Deplano A., Vandendriessche S., Bolfíková B. Černá, Hulva P., Arendrup M.C., Hare R.K., Barnadas C., Stegger M., Sieber R.N., Skov R.L., Petersen A., Angen Ø., Rasmussen S.L., Espinosa-Gongora C., Aarestrup F.M., Lindholm L.J., Nykäsenoja S.M., Laurent F., Becker K., Walther B., Kehrenberg C., Cuny C., Layer F., Werner G., Witte W., Stamm I., Moroni P., Jørgensen H.J., de Lencastre H., Cercenado E., García-Garrote F., Börjesson S., Hæggman S., Perreten V., Teale C.J., Waller A.S., Pichon B., Curran M.D., Ellington M.J., Welch J.J., Peacock S.J., Seilly D.J., Morgan F.J.E., Parkhill J., Hadjirin N.F., Lindsay J.A., Holden M.T.G., Edwards G.F., Foster G., Paterson G.K., Didelot X., Holmes M.A., Harrison E.M., Larsen A.R. (2022). Emergence of methicillin resistance predates the clinical use of antibiotics. Nature.

[14] Bengtsson B., Persson L., Ekström K., Unnerstad H.E., Uhlhorn H., Börjesson S. (2017). High occurrence of mecC -MRSA in wild hedgehogs (Erinaceus europaeus) in Sweden. Vet. Microbiol..

[15] Dube F., Söderlund R., Lampinen Salomonsson M., Troell K., Börjesson S. (2021). Benzylpenicillin-producing Trichophyton erinacei and methicillin resistant Staphylococcus aureus carrying the mecC gene on European hedgehogs – A pilot-study. BMC Microbiol..

[16] Smith J.M.B., Marples M.J. (1965). Dermatophyte lesions in the hedgehog as a reservoir of penicillin-resistant staphylococci. J. Hyg..

[17] Laborda P., Sanz-García F., Ochoa-Sánchez L.E., Gil-Gil T., Hernando-Amado S., Martínez J.L. (2022). Wildlife and antibiotic resistance. Front. Cell. Infect. Microbiol..

[18] Atterby C., Börjesson S., Ny S., Järhult J.D., Byfors S., Bonnedahl J. (2017). ESBL-producing Escherichia coli in Swedish gulls—A case of environmental pollution from humans?. PLoS One.

[19] Checcucci A., Trevisi P., Luise D., Modesto M., Blasioli S., Braschi I., Mattarelli P. (2020). Exploring the animal waste Resistome: the spread of antimicrobial resistance genes through the use of livestock manure. Front. Microbiol..

[20] Worsley-Tonks K.E.L., Miller E.A., Anchor C.L., Bender J.B., Gehrt S.D., McKenzie S.C., Singer R.S., Johnson T.J., Craft M.E. (2021). Importance of anthropogenic sources at shaping the antimicrobial resistance profile of a peri-urban mesocarnivore. Sci. Total Environ..

[21] Garcias B., Aguirre L., Seminati C., Reyes N., Allepuz A., Obón E., Molina-Lopez R.A., Darwich L. (2021). Extended-Spectrum β-lactam resistant Klebsiella pneumoniae and Escherichia coli in wild European hedgehogs (Erinaceus europeus) living in populated areas. Animals..

[22] EUCAST (2022). Antimicrobial Wild Type Distributions of Microorganisms. https://mic.eucast.org.

[23] Stegger M., Andersen P.S., Kearns A., Pichon B., Holmes M.A., Edwards G., Laurent F., Teale C., Skov R., Larsen A.R. (2012). Rapid detection, differentiation and typing of methicillin-resistant Staphylococcus aureus harbouring either mecA or the new mecA homologue mecALGA251. Clin. Microbiol. Infect..

[24] EURL-AR (2012). Protocol for PCR Amplification af mecA, mecC (mecALGA251), spa and PVL, 2nd version. https://www.eurl-ar.eu/CustomerData/Files/Folders/21-protocols/279_pcr-spa-pvl-meca-mecc-sept12.pdf.

[25] Shopsin B., Gomez M., Montgomery S.O., Smith D.H., Waddington M., Dodge D.E., Bost D.A., Riehman M., Naidich S., Kreiswirth B.N. (1999). Evaluation of protein A gene polymorphic region DNA sequencing for typing of *Staphylococcus aureus* strains. J. Clin. Microbiol..

[26] Jünemann S., Sedlazeck F.J., Prior K., Albersmeier A., John U., Kalinowski J., Mellmann A., Goesmann A., von Haeseler A., Stoye J., Harmsen D. (2013). Updating benchtop sequencing performance comparison. Nat. Biotechnol..

[27] Ewels P., Magnusson M., Lundin S., Käller M. (2016). MultiQC: summarize analysis results for multiple tools and samples in a single report. Bioinformatics..

[28] Bolger A.M., Lohse M., Usadel B. (2014). Trimmomatic: a flexible trimmer for Illumina sequence data. Bioinformatics..

[29] Bankevich A., Nurk S., Antipov D., Gurevich A.A., Dvorkin M., Kulikov A.S., Lesin V.M., Nikolenko S.I., Pham S., Prjibelski A.D., Pyshkin A.V., Sirotkin A.V., Vyahhi N., Tesler G., Alekseyev M.A., Pevzner P.A. (2012). SPAdes: A new genome assembly algorithm and its applications to single-cell sequencing. J. Comput. Biol..

[30] Bortolaia V., Kaas R.S., Ruppe E., Roberts M.C., Schwarz S., Cattoir V., Philippon A., Allesoe R.L., Rebelo A.R., Florensa A.F., Fagelhauer L., Chakraborty T., Neumann B., Werner G., Bender J.K., Stingl K., Nguyen M., Coppens J., Xavier B.B., Malhotra-Kumar S., Westh H., Pinholt M., Anjum M.F., Duggett N.A., Kempf I., Nykäsenoja S., Olkkola S., Wieczorek K., Amaro A., Clemente L., Mossong J., Losch S., Ragimbeau C., Lund O., Aarestrup F.M. (2020). ResFinder 4.0 for predictions of phenotypes from genotypes. J. Antimicrob. Chemother..

[31] Carattoli A., Zankari E., García-Fernández A., Voldby Larsen M., Lund O., Villa L., Møller Aarestrup F., Hasman H. (2014). In silico detection and typing of plasmids using PlasmidFinder and plasmid multilocus sequence typing. Antimicrob. Agents Chemother..

[32] Joensen K.G., Scheutz F., Lund O., Hasman H., Kaas R.S., Nielsen E.M., Aarestrup F.M. (2014). Real-time whole-genome sequencing for routine typing, surveillance, and outbreak detection of Verotoxigenic Escherichia coli. J. Clin. Microbiol..

[33] Kaya H., Hasman H., Larsen J., Stegger M., Johannesen T.B., Allesøe R.L., Lemvigh C.K., Aarestrup F.M., Lund O., Larsen A.R. (2018). SCCmec finder, a web-based tool for typing of staphylococcal cassette chromosome mec in *Staphylococcus aureus* using whole-genome sequence data. MSphere..

[34] Larsen M., Cosentino S., Rasmussen S., Friis C., Hasman H., Marvig R.L., Jelsbak L., Sicheritz-Pontén T., Ussery D., Aarestrup F., Lund O. (2012). Multilocus sequence typing of Total-genome-sequenced bacteria. J. Clin. Microbiol..

[35] van Wamel W.J.B., Rooijakkers S.H.M., Ruyken M., van Kessel K.P.M., van Strijp J.A.G. (2006). The innate immune modulators staphylococcal complement inhibitor and chemotaxis inhibitory protein of *Staphylococcus aureus* are located on β-Hemolysin-converting bacteriophages. J. Bacteriol..

[36] Jolley K.A., Bray J.E., Maiden M.C.J. (2018). Open-access bacterial population genomics: BIGSdb software, the PubMLST.org website and their applications. Wellcome Open Res..

[37] Zerbino D.R., Birney E. (2008). Velvet: algorithms for de novo short read assembly using de Bruijn graphs. Genome Res..

[38] Leopold S.R., Goering R.V., Witten A., Harmsen D., Mellmann A. (2014). Bacterial whole-genome sequencing revisited: portable, scalable, and standardized analysis for typing and detection of virulence and antibiotic resistance genes. J. Clin. Microbiol..

[39] Rautio A., Valtonen A., Kunnasranta M. (2013). The effects of sex and season on home range in European hedgehogs at the northern edge of the species range. Ann. Zool. Fenn..

[40] Bartels M.D., Worning P., Andersen L.P., Bes M., Enger H., Ås C.G., Hansen T.A., Holzknecht B.J., Larssen K.W., Laurent F., Mäkitalo B., Pichon B., Svartström O., Westh H. (2021). Repeated introduction and spread of the MRSA clone t304/ST6 in northern Europe. Clin. Microbiol. Infect..

[41] Petersen A., Larssen K.W., Gran F.W., Enger H., Hæggman S., Mäkitalo B., Haraldsson G., Lindholm L., Vuopio J., Henius A.E., Nielsen J., Larsen A.R. (2021). Increasing incidences and clonal diversity of methicillin-resistant *Staphylococcus aureus* in the Nordic countries - results from the Nordic MRSA surveillance. Front. Microbiol..

[42] Pauly N., Wichmann-Schauer H., Ballhausen B., Torres Reyes N., Fetsch A., B.-A. (2019). Tenhagen, detection and quantification of methicillin-resistant *Staphylococcus aureus* in fresh broiler meat at retail in Germany. Int. J. Food Microbiol..

[43] Kalupahana R.S., Duim B., Verstappen K.M., Gamage C.D., Dissanayake N., Ranatunga L., Graveland H., Wagenaar J.A. (2019). MRSA in pigs and the environment as a risk for employees in pig-dense areas of Sri Lanka. Front. Sustain. Food Syst..

[44] Ge M., Zayda Y. Masuda, Hammad A.M., Honjoh K., Elbagory A.M., Miyamoto T. (2020). Molecular characterisation of methicillin-resistant (MRSA) and methicillin-susceptible (MSSA) *Staphylococcus aureus* isolated from bovine subclinical mastitis and Egyptian raw milk cheese. Int. Dairy J..

[45] Bierowiec K., Płoneczka-Janeczko K., Rypuła K. (2016). Is the colonisation of Staphylococcus aureus in pets associated with their close contact with owners?. PLoS One.

[46] Chen Q., Xie S. (2019). Genotypes, enterotoxin gene profiles, and antimicrobial resistance of Staphylococcus aureus associated with foodborne outbreaks in Hangzhou, China. Toxins (Basel)..

[47] Ruiz-Ripa L., Alcalá L., Simón C., Gómez P., Mama O.M., Rezusta A., Zarazaga M., Torres C. (2019). Diversity of Staphylococcus aureus clones in wild mammals in Aragon, Spain, with detection of MRSA ST130- *mecC* in wild rabbits. J. Appl. Microbiol..

[48] Sahin-Tóth J., Albert E., Juhász A., Ghidán Á., Juhász J., Horváth A., Steward M.C., Dobay O. (2022). Prevalence of Staphylococcus aureus in wild hedgehogs (Erinaceus europaeus) and first report of mecC-MRSA in Hungary. Sci. Total Environ..

[49] Gindonis V., Taponen S., Myllyniemi A.-L., Pyörälä S., Nykäsenoja S., Salmenlinna S., Lindholm L., Rantala M. (2013). Occurrence and characterization of methicillin-resistant staphylococci from bovine mastitis milk samples in Finland. Acta Vet. Scand..

[50] Dupieux C., Kolenda C., Larsen A.R., Pichon B., Holmes M., Bes M., Teale C., Dickson E., Hill R., Skov R., Kearns A., Laurent F. (2017). Variable performance of four commercial chromogenic media for detection of methicillin-resistant Staphylococcus aureus isolates harbouring mecC. Int. J. Antimicrob. Agents.

[51] English M.P., Morris P. (1969). Trichophyton mentagrophytes var. erinacei in hedgehog nests. Med. Mycol..

