# Supplementary material for: Clonal dissemination of successful emerging clone mecA-MRSA t304/ST6 among humans and hedgehogs in the Helsinki metropolitan area in Finland
Source: One Health. 2023 Feb 21;16:100516. doi: 10.1016/j.onehlt.2023.100516 (PMC10288059; doi:10.1016/j.onehlt.2023.100516)
Supplement: Supplementary Table 1 — Background information for all the studied hedgehogs (n = 115). [file mmc2.docx]

Supplementary table 1 Background information for all the studied hedgehogs (n = 115).

| ID | Number of isolates selected for species confirmation | ^[[1]](#endnote-1)^Species confirmation (Maldi-tof)^[[2]](#endnote-2)^ | Date of admission to the wildlife hospital | Date of death/euthanasia | City | Residential district | Time spent in the hospital (d) | Reason for being admitted to the hospital | Cause of death | Sex | Age | Weight (g) |
| --- | --- | --- | --- | --- | --- | --- | --- | --- | --- | --- | --- | --- |
| D1155 | 1 | *Enterococcus faecalis* | 26.8.2020 | 26.8.2020 | Helsinki |  |  | Died immediately upon arrival | Deceased | Male | Juvenile |  |
| D252 | 1 | *Enterococcus faecium* | 27.5.2020 | 27.5.2020 | Vantaa |  | 0 | Bite wounds, dyspnea | Euthanized | Male | Adult |  |
| D774 | 1 | *Enterococcus faecium* | 14.7.2020 | 15.7.2020 | Tuusula |  | 1 | Fractured leg | Euthanized | Male | Adult |  |
| D873 | 2 | *Enterococcus faecium* | 24.7.2020 | 24.7.2020 | Helsinki |  | 0 | Emaciated, fractured hindlegs | Euthanized | Male | Juvenile |  |
| D930 | 1 | *Enterococcus faecium* | 30.7.2020 | 30.7.2020 | Helsinki |  |  | Stuck in a floorball net, maggot infestation | Deceased | Male | Juvenile |  |
| D1068 | 1 | *Enterococcus faecium* | 12.8.2020 | 12.8.2020 | Helsinki |  | 0 | Open fracture in hindleg, maggot infestation | Euthanized | Female | Adult |  |
| D1111 | 1 | *Enterococcus faecium* | 19.8.2020 | 19.8.2020 | Helsinki |  | 0 | Neurological symptoms, circling, apathy, maggot infestation | Euthanized | Female | Juvenile |  |
| D1234 | 1 | *Enterococcus faecium* | 12.9.2020 | 16.9.2020 | Riihimäki |  | 4 | Front leg injury | Euthanized | Male | Adult | 1430 |
| D26012021/1 | 2 | *Enterococcus faecium* |  |  |  |  |  |  |  | Male |  |  |
| D935 | 1 | *Enterococcus gallinarum* | 30.7.2020 | 30.7.2020 | Helsinki |  | 0 | Front paw stuck on mouse trap, maggot infestation | Euthanized | Male | Juvenile |  |
| D1029 | 1 | *Enterococcus hirae* | 8.8.2020 | 8.8.2020 | Helsinki |  | 0 | Right hindleg fractured, part of the foot missing | Euthanized | Male | Adult |  |
| D351 | 2 | *Lycinibacillus fusiformis, Enterococcus faecalis* | 7.6.2020 | 8.6.2020 | Helsinki |  | 1 | Exhausted | Deceased | Female | Adult | 690 |
| D679 | 1 | No organism identification possible | 5.7.2020 | 5.7.2020 | Vantaa |  | 0 | Fractured leg | Euthanized | Male | Adult |  |
| D5 | 1 | *Staphylococcus aureus* | 22.4.2020 | 22.4.2020 | Helsinki | Munkkivuori | 0 | Fractured hindlegs | Euthanized | Male | Adult | > 1000 |
| D4 | 1 | *Staphylococcus aureus* | 15.5.2020 | 15.5.2020 | Helsinki | Malmi | 0 | Fractured leg | Euthanized |  | Adult |  |
| D3 | 1 | *Staphylococcus aureus* | 16.5.2020 | 16.5.2020 | Helsinki | Vuosaari |  | Not moving, flank mass | Deceased |  | Adult |  |
| D6 | 1 | *Staphylococcus aureus* | 22.5.2020 | 23.5.2020 | Helsinki | Malmi | 1 | Dyspnea | Euthanized |  | Adult |  |
| D1 | 1 | *Staphylococcus aureus* | 23.5.2020 | 23.5.2020 | Vantaa | Louhela | 0 | Neurological symptoms, abnormal posture | Euthanized |  | Adult | 564 |
| D8 | 1 | *Staphylococcus aureus* | 24.5.2020 | 28.5.2020 | Helsinki | Etu-Töölö | 4 | Neurological symptoms, circling | Euthanized |  | Adult | 695 |
| D411 | 1 | *Staphylococcus aureus* | 12.6.2020 | 23.6.2020 | Helsinki | Meilahti | 10 | Neurological symptoms | Euthanized | Male | Adult |  |
| D884 | 1 | *Staphylococcus aureus* | 26.7.2020 | 26.7.2020 | Helsinki | Landbo | 0 | Orphan | Euthanized | Female | Juvenile |  |
| D2 | 1 | *Staphylococcus aureus* |  |  | Kirkkonummi | Kuusela |  |  |  |  |  |  |
| D7 | 1 | *Staphylococcus aureus* |  |  | - | - |  |  |  |  |  |  |
| D888 | 2 | *Staphylococcus aureus, Staphylococcus sciuri* | 26.7.2020 | 26.7.2020 | Helsinki | Etu-Töölö | 0 | Ocular luxation | Euthanized | Male | Juvenile |  |
| D743 | 2 | *Staphylococcus sciuri, Enterococcus faecium* |  |  |  |  |  | Abdominal wound, sarcoptic mange |  | Female |  |  |
| D114 | 1 | *Staphylococcus simulans* |  |  |  |  |  |  |  |  |  |  |
| D948 | 1 | *Staphylococcus simulans* |  |  |  |  |  |  |  | Male |  |  |
| D008 |  |  | 8.1.2020 | 26.1.2020 | Helsinki |  | 18 | Emaciated | Deceased |  | Adult | 600 |
| D50 |  |  | 15.3.2020 | 15.3.2020 | Helsinki |  | 0 | Bite wounds, other hindleg missing | Euthanized |  | Adult | 886 |
| D77 |  |  | 9.4.2020 | 11.4.2020 | Helsinki |  | 2 | Front paw missing | Euthanized |  | Adult | 483 |
| D86 |  |  | 14.4.2020 | 14.4.2020 | Helsinki |  | 0 | Cranial fracture | Euthanized | Male | Adult | 450 |
| D95 |  |  | 20.4.2020 | 20.4.2020 | Helsinki |  | 0 | Traumatic wound on face | Euthanized |  | Adult |  |
| D100 |  |  | 21.4.2020 | 23.4.2020 | Helsinki |  | 2 | Paraplegia | Euthanized | Male | Adult | 482 |
| D116 |  |  | 27.4.2020 | 28.4.2020 | Vantaa |  | 1 | Hemoptysis, weak | Euthanized |  | Adult | 880 |
| D153 |  |  | 8.5.2020 | 8.5.2020 | Helsinki |  | 0 | Ocular perforation | Euthanized | Male | Adult |  |
| D166 |  |  | 10.5.2020 | 11.5.2020 | Helsinki |  | 1 | Leg stuck on rat trap | Euthanized |  | Adult |  |
| D167 |  |  | 11.5.2020 | 11.5.2020 | Helsinki |  | 0 | Open fracture in leg | Euthanized |  | Adult |  |
| D177 |  |  | 12.5.2020 | 12.5.2020 | Helsinki |  | 0 | Hip luxation | Euthanized | Female | Adult | 465 |
| D179 |  |  | 13.5.2020 | 13.5.2020 | Helsinki |  | 0 | Traumatic found on forehead | Euthanized | Male | Adult |  |
| D209 |  |  | 19.5.2020 | 19.5.2020 | Helsinki |  |  | Injured | Dead on arrival |  | Adult |  |
| D221 |  |  | 22.5.2020 | 22.5.2020 | Espoo |  | 0 | Hindleg wounded and swollen | Euthanized |  | Adult |  |
| D248 |  |  | 26.5.2020 | 26.5.2020 | Helsinki |  |  | Missing toes, infected leg | Dead on arrival |  | Adult |  |
| D472 |  |  | 18.6.2020 | 18.6.2020 | Sipoo |  | 0 | Left hindleg swollen | Euthanized | Male | Adult |  |
| D543 |  |  | 23.6.2020 | 23.6.2020 | Helsinki |  | 0 | Half of the face missing | Euthanized | Female | Adult |  |
| D582 |  |  | 26.6.2020 | 26.6.2020 | Espoo |  | 0 | Facial bite wound | Euthanized | Female | Adult |  |
| D731 |  |  | 9.7.2020 | 9.7.2020 | Helsinki |  | 0 | Face missing, fractured right hindleg | Euthanized | Male | Juvenile |  |
| D764 |  |  | 13.7.2020 | 13.7.2020 | Helsinki |  | 0 | Fractured maxilla, gas gangrene | Euthanized |  | Juvenile |  |
| D779 |  |  | 14.7.2020 | 15.7.2020 | Espoo |  | 1 | Stuck on mouse trap | Euthanized | Male | Juvenile |  |
| D854 |  |  | 22.7.2020 | 26.7.2020 | Tuusula |  | 4 | Emaciated, dehydrated | Euthanized |  | Adult |  |
| D947 |  |  | 31.7.2020 | 31.7.2020 | Helsinki |  |  | Orphan, maggot infestation | Deceased | Female | Juvenile | 178 |
| D957 |  |  | 1.8.2020 | 2.8.2020 | Helsinki |  | 1 | Orphan | Deceased | Male | Juvenile | 184 |
| D963 |  |  | 1.8.2020 | 1.8.2020 | Helsinki |  | 0 | Open wound on back, maggot infestation | Euthanized | Male | Adult |  |
| D1028 |  |  | 8.8.2020 | 8.8.2020 | Helsinki |  | 0 | Maggot infestation | Euthanized | Male | Juvenile |  |
| D1064 |  |  | 11.8.2020 | 11.8.2020 | Helsinki |  | 0 | Infected bite wounds | Euthanized | Female | Adult |  |
| D1152 |  |  | 26.8.2020 | 26.8.2020 | Helsinki |  |  | Maggot infestation | Deceased | Female | Juvenile |  |
| D1246 |  |  | 14.9.2020 | 21.9.2020 | Espoo |  | 7 | Trauma caused by a lawn mower | Euthanized | Male | Adult | 1140 |
| D1440 |  |  | 18.11.2020 | 22.11.2020 | Espoo |  | 4 | Emaciated, dehydrated, sarcoptic mange | Deceased | Male | Adult | 689 |
| D1487 |  |  | 25.12.2020 | 26.12.2020 | Helsinki |  | 1 | Nasal trauma | Euthanized | Female | Adult | 513 |
| D079 |  |  | 9.5.2021 | 17.5.2021 | Helsinki |  | 8 | Hemiplegia, wounded | Euthanized | Male | Adult | 627 |
| D113 |  |  | 23.5.2021 | 24.5.2021 | Helsinki |  | 1 | Paralyzed, swollen face | Deceased | Male | Adult | 581 |
| D129 |  |  | 28.5.2021 | 28.5.2021 | Espoo |  | 0 | Ocular perforation, pecked by a bird | Euthanized | Female | Adult |  |
| D156 |  |  | 4.6.2021 | 5.6.2021 | Helsinki |  | 1 | Dehydrated | Deceased | Female | Adult |  |
| D160 |  |  | 5.6.2021 | 5.6.2021 | Helsinki |  | 1 | Pecked by a bird, facial wound | Euthanized | Male | Adult |  |
| D266 |  |  | 24.6.2021 | 24.6.2021 | Helsinki |  | 0 | Paraplegia | Euthanized | Male | Adult |  |
| D358 |  |  | 8.7.2021 | 8.7.2021 | Espoo |  | 0 | Orphan, hypothermia, maggot infestation | Euthanized | Male | Juvenile | 638 |
| D357 |  |  | 8.7.2021 | 8.7.2021 | Vantaa |  | 0 | Fractured ankle, infected and swollen | Euthanized | Male | Adult |  |
| D374 |  |  | 10.7.2021 | 10.7.2021 | Helsinki |  | 0 | Dyspnea | Euthanized | Male | Adult |  |
| D449 |  |  | 16.7.2021 | 24.7.2021 | Sipoo |  | 8 | Cloudy cornea, circling, otitis externa | Euthanized | Male | Adult |  |
| D471 |  |  | 18.7.2021 | 5.8.2021 | Helsinki |  | 18 | Orphan | Deceased | Female | Juvenile |  |
| D479 |  |  | 18.7.2021 | 18.7.2021 | Helsinki |  | 0 | Gas gangrene, facial wound, anal wound, maggot infestation, dyspnea | Euthanized | Male | Juvenile |  |
| D482 |  |  | 19.7.2021 | 19.7.2021 | Tuusula |  | 0 | Infected wound on neck, maggot infestation, right hindleg fracture | Euthanized | Female | Adult |  |
| D484 |  |  | 19.7.2021 | 4.8.2021 | Helsinki |  | 16 | Orphan | Deceased | Female | Juvenile |  |
| D521 |  |  | 21.7.2021 | 24.7.2021 | Helsinki |  | 3 | Orphan | Deceased | Female | Juvenile |  |
| D511 |  |  | 21.7.2021 | 21.7.2021 | Helsinki |  | 0 | Trauma caused by a lawn mower, fractured leg | Euthanized | Female | Juvenile |  |
| D527 |  |  | 22.7.2021 | 23.7.2021 | Helsinki |  | 1 | Orphan | Deceased | Female | Juvenile |  |
| D561 |  |  | 24.7.2021 | 5.8.2021 | Helsinki |  | 12 | Injured, bleeding | Euthanized | Female | Adult | 718 |
| D586 |  |  | 25.7.2021 | 26.7.2021 | Helsinki |  | 1 | Orphan | Deceased | Male | Juvenile |  |
| D576 |  |  | 25.7.2021 | 29.7.2021 | Helsinki |  | 4 | Exhausted, bloody discharge on face | Euthanized | Male | Juvenile | 282 |
| D593 |  |  | 26.7.2021 | 26.7.2021 | Vantaa |  | 0 | Deep wound, maggot infestation | Euthanized | Male | Juvenile |  |
| D628 |  |  | 28.7.2021 | 31.7.2021 | Espoo |  | 3 | Bilateral eye infection, gas gangrene | Euthanized | Male | Adult |  |
| D668 |  |  | 31.7.2021 | 1.8.2021 | Helsinki |  | 1 | Orphan | Euthanized | Female | Juvenile |  |
| D663 |  |  | 31.7.2021 | 31.7.2021 | Hyvinkää |  | 0 | Injured hindleg, maggot infestation | Euthanized | Male | Immature |  |
| D685 |  |  | 2.8.2021 | 2.8.2021 | Sipoo |  | 0 | Fractured hindleg | Euthanized | Male | Adult |  |
| D726 |  |  | 5.8.2021 | 5.8.2021 | Espoo |  | 0 | Mandibular fracture, dyspnea | Euthanized | Male | Adult |  |
| D738 |  |  | 6.8.2021 | 9.8.2021 | Helsinki |  | 3 |  | Deceased | Male | Juvenile |  |
| D742 |  |  | 6.8.2021 | 12.8.2021 | Sipoo |  | 6 | Bitten by a dog | Deceased | Female | Adult |  |
| D736 |  |  | 6.8.2021 | 23.8.2021 | Espoo |  | 17 |  | Euthanized | Female | Immature |  |
| D745 |  |  | 8.8.2021 | 23.8.2021 | Helsinki |  | 14 | Ear abscess | Deceased | Female | Juvenile |  |
| D786 |  |  | 11.8.2021 | 13.8.2021 | Helsinki |  | 2 |  | Deceased | Female | Juvenile |  |
| D810 |  |  | 13.8.2021 | 13.8.2021 | Helsinki |  | 0 | Fractured leg | Euthanized | Male | Immature |  |
| D835 |  |  | 16.8.2021 | 17.8.2021 | Vantaa |  | 1 | Maggot infestation | Deceased | Male | Immature | 286 |
| D878 |  |  | 21.8.2021 | 21.8.2021 | Helsinki |  | 0 | Right shoulder dislocation, ocular perforation | Euthanized | Female | Immature | 477 |
| D887 |  |  | 22.8.2021 | 23.8.2021 | Nurmijärvi |  | 1 | Sick | Deceased | Female | Immature | 224 |
| D890 |  |  | 23.8.2021 | 23.8.2021 | Helsinki |  | 0 | Mandibular trauma/wound | Euthanized | Male | Adult |  |
| D20 |  |  |  |  |  |  |  |  |  |  |  |  |
| D265 |  |  |  |  |  |  |  |  |  |  |  |  |
| D151 |  |  |  |  |  |  |  |  |  |  |  |  |
| D174 |  |  |  |  |  |  |  |  |  |  |  |  |
| D636-20a |  |  |  |  |  |  |  |  |  |  |  |  |
| D1414 |  |  |  |  |  |  |  |  |  | Female |  |  |
| D603 |  |  |  |  |  |  |  |  |  | Female |  |  |
| D26012021/2 |  |  |  |  |  |  |  |  |  |  |  |  |
| D1609-21a |  |  |  |  |  |  |  |  |  | Male |  |  |
| D1609-21b |  |  |  |  |  |  |  |  |  | Female |  |  |
| D1608 |  |  |  |  |  |  |  |  |  | Male |  |  |
| D1508 |  |  |  |  |  |  |  |  |  | Female |  |  |
| D2607 |  |  |  |  |  |  |  |  |  | Male |  |  |
| D1609-21c |  |  |  |  |  |  |  |  |  | Male |  |  |
| D1008 |  |  |  |  |  |  |  |  |  | Male |  |  |
| D0808 |  |  |  |  |  |  |  |  |  | Female |  |  |
| D217 |  |  |  |  |  |  |  |  |  | Female |  |  |
| D1582021 |  |  |  |  |  |  |  |  |  | Male |  |  |
| D1982021 |  |  |  |  |  |  |  |  |  | Male | Juvenile |  |
| D317 |  |  |  |  |  |  |  |  |  | Female | Juvenile |  |

1. [↑](#endnote-ref-1)
2. Matrix-assisted laser-desorption-ionization time-of-flight [↑](#endnote-ref-2)
